# Binocular function in patients with intermittent exotropia accompanied by unilateral congenital ptosis

**DOI:** 10.1038/s41598-022-23254-1

**Published:** 2022-10-31

**Authors:** Hee-young Choi, Su-Jin Kim, Sang-Yoon Kim, Jung Hyo Ahn, Ji-Eun Lee

**Affiliations:** 1 Department of Ophthalmology, Pusan National University KR, Biomedical Research Institute, Pusan National University Hospital, Busan, Korea; 2 Department of Ophthalmology, Pusan National University School of Medicine, Research Institute for Convergence of Biomedical Science and Technology, Pusan National University Yangsan Hospital, Yangsan, 50612 South Korea; 3 Department of Ophthalmology, Stanford University School of Medicine, Palo Alto, CA USA

**Keywords:** Eyelid diseases, Eye abnormalities

## Abstract

Patients with intermittent exotropia (IXT) have a wide range of binocular deficits. This study aims to evaluate the effect of ptosis on the binocular function of patients with IXT. Clinical records of 45 IXT patients with congenital ptosis (IXT-ptosis group) and 58 age-matched IXT patients without ptosis (IXT only group) who presented for eye examination between January 2017 and June 2020 were retrospectively reviewed. Patients with amblyopia were excluded to rule out the effects of visual acuity on binocularity. Best-corrected visual acuity (BCVA), the magnitude of exodeviation at distance and at near, stereopsis, and office-based control scores at the first visit were reviewed. The binocular functions of the two groups were compared. The mean ± SD age of the overall patients was 6.6 ± 2.7 years. There were no significant differences in the distribution of age, sex, spherical equivalent refraction, or BCVA between the two groups (all *p* > 0.05). Although the office-based control scores at distance and near were slightly worse in the IXT-ptosis group, the differences were not statistically significant (at distance, 2.8 ± 1.87 vs. 2.2 ± 1.13, *p* = 0.08; at near, 1.8 ± 0.67 vs. 1.6 ± 0.74, *p* = 0.11). Furthermore, the IXT-ptosis group had worse stereopsis at distance (*p* = 0.01). There were no significant differences between the two groups in near stereopsis or exodeviation magnitude (*p* > 0.05). A larger proportion of patients had suppression on the Bagolini test in the IXT-ptosis group than in the IXT-only group (*p* = 0.04). The IXT-ptosis group had worse distance stereoacuity, and a larger proportion of patients had suppression on the Bagolini test than the IXT only group. In IXT patients, the presence of coexisting ptosis can have a further deleterious impact on binocular function.

## Introduction

Strabismus has an estimated prevalence of 1 to 5% in the general population^1,2^. It has been reported that the prevalence of strabismus is 10.3 to 32% in patients with congenital ptosis, which is at least four times higher than the prevalence in the general population^3–5^. Genetic predisposition, intrauterine insult to the overlapping regions of the oculomotor nuclear complex or third cranial nerve problems might play a role in the pathogegesis of strabismus; additionally, some cases might be caused by visual occlusion and disruption of binocularity by the ptotic eyelid^4–6^.

Intermittent exotropia (IXT) is characterized by periods of normal binocular alignment with sensory fusion and manifestation of exotropia at other times. This accounts for the majority of exotropia reported worldwide^7^. IXT is the most common form of strabismus in Korean children, with an estimated prevalence of 10.5%, which is 6.4 times more common than esodeviation^8^. Systemic conditions such as fatigue or general illness may exacerbate the angle or control of this deviation. Decorrelated binocular inputs impede the normal development of binocular vision, during misalignment. Correlated binocular inputs during alignment can promote the development of binocular vision. Therefore, IXT patients have an understandably large range of binocular deficits, ranging from poor binocular fusion to normal stereopsis^9^. The ptotic eyelid can occlude visual input and may disrupt control or fusion. Binocular function can be further affected when ptosis coexists with IXT.

Although studies have examined the prevalence and types of strabismus among children with congenital ptosis^3–5^, the characteristics of IXT coexisting with congenital ptosis compared to strabismus without ptosis have not yet been reported. Thus, the purpose of this study was to compare binocular functions between IXT patients with unilateral congenital ptosis and those without ptosis.

## Results

The mean ± standard deviation (SD) age of the patients was 6.6 ± 2.7 years. There were 53 males and 50 females in the cohort. Of these 103 patients, 45 had IXT with unilateral congenital ptosis and 58 did not have congenital ptosis. There was no statistically significant difference in age, sex, refractive errors or BCVA between the two groups. The cylinder in both eyes was slightly larger in the IXT-ptosis group than in the IXT-only group, but the differences were not significant (*p* = 0.07 and 0.09, respectively) (Table 1).

**Table 1:** Baseline characteristics and clinical features : comparison between the IXT-ptosis and IXT-only groups.

	IXT-ptosis (n = 45)	IXT-only (n = 58)	*p *value
Age (years, mean ± SD)	6.5 ± 2.9	6.7 ± 2.0	0.45
Sex (M:F)	26:19	31:27	0.12
Cycloplegic refraction, OD (D, mean ± SD)			
Spherical	−0.67 ± 2.57	−0.42 ± 1.99	0.29
Cylinder	−1.28 ± 1.05	−0.75 ± 0.86	0.07
Spherical equivalent	−1.31 ± 2.84	−0.80 ± 1.93	0.17
Cycloplegic refraction, OS (D, mean ± SD)			
Spherical	−0.12 ± 1.67	−0.33 ± 1.94	0.34
Cylinder	−1.52 ± 1.06	−0.93 ± 0.88	0.09
Spherical equivalent	−0.90 ± 2.05	−0.79 ± 1.97	0.67
BCVA (logMAR, mean ± SD)			
Right eye	0.06 ± 0.08	0.03 ± 0.07	0.42
Left eye	0.02 ± 0.06	0.04 ± 0.08	0.47
Magnitude of exodeviation (PD, mean ± SD)			
At distance	20.4 ± 7.7	25.6 ± 8.7	0.39
At near	22.5 ± 7.3	25.8 ± 9.7	0.41
Stereopsis (log arcsec, mean ± SD)			
Titmus test	2.24 ± 0.71	1.99 ± 0.55	0.12
Randot Preschool Stereo test	2.01 ± 0.35	1.86 ± 0.34	0.19
Distance stereo test	2.85 ± 0.53	2.21 ± 0.50	0.02
Presence of suppression, n (%)			
4PD base out test	20 (44.4)	16 (27.6)	0.25
Bagolini test	8 (17.8)	2 (3.5)	0.04
Worth 4 dot test	14 (31.1)	12 (20.7)	0.38
Office based control score (mean ± SD)			
At distance	2.8 ± 1.87	2.2 ± 1.13	0.08
At near	1.8 ± 0.67	1.6 ± 0.74	0.11
Constant exotropia, n (%)	5 (11.1)	5 (8.6)	0.75
Oblique muscle overaction, n (%)	16 (35.5)	19 (32.8)	0.65
Dissociated vertical deviation, n (%)	4 (8.9)	5 (8.6)	0.88
Coexisting vertical strabismus, n (%)	3 (6.7)	3 (5.2)	0.83
Latent nystagmus, n (%)	1 (2.2)	0 (0)	> 0.99

*BCVA* best-corrected visual acuity, *D* dioptre, *IXT* intermittent exotropia, *MAR* minimum angle of resolution, *PD* prism diopters, *SD* standard deviation.

The magnitudes of exodeviation at distance and near did not differ significantly between the two groups (at distance, 20.4 ± 7.7 vs. 25.6 ± 8.7, *p* = 0.39; at near, 22.5 ± 7.3 vs. 25.8 ± 9.7, *p* = 0.41). The mean distance stereoacuity, which was measured using the Distance Randot test, was significantly worse in the IXT-ptosis group (2.85 ± 0.53 log arcsec in the IXT-ptosis group, 2.21 ± 0.50 log arcsec in the IXT only group, *p* = 0.02). Near stereoacuity, which was measured using the Randot stereo-test and the Titmus test, did not differ significantly between the two groups (Randot stereo-test, 2.01 ± 0.35 vs. 1.86 ± 0.34, *p* = 0.19; Titmus test, 2.24 ± 0.74 vs. 1.99 ± 0.55, *p* = 0.12). The presence of suppression based on the 4-prism dioptre base-out test and the Worth 4 Dot test did not differ significantly. However, the number of patients with suppression based on the Bagolini test was larger in the IXT-ptosis group than in the IXT-only group (*p* = 0.04). Although the office-based control scores at distance and near were slightly worse in the IXT-ptosis group, the differences were not statistically significant (*p* = 0.08 and *p* = 0.11, respectively) (Table 1).

The number of patients with constant exotropia at distance, inferior oblique overaction, dissociated vertical deviation, and coexisting vertical strabismus were likewise not significantly different between the two groups (constant exotropia, 5 (11.1%) vs. 5 (8.6%), *p* = 0.75; oblique muscle overaction, 16 (35.5%) vs. 19 (32.8%), *p* = 0.65; dissociated vertical deviation, 4 (8.9%) vs. 5 (8.6%), *p* = 0.88; coexisting vertical strabismus, 3 (6.7%) vs. 3 (5.2%), *p* = 0.83) (Table 1).

There were no patients with MRD1 ≤ 0. The magnitudes of exodeviation, office-based control scores and near stereopsis did not differ significantly between the 0 < MRD1 ≤ 1 group and the 1 < MRD1 ≤ 2 group. Although distance stereopsis tended to be better in the 1 < MRD1 ≤ 2 group than in the 1 < MRD1 ≤ 2 group, the difference between the two groups was not significant (*p* = 0.07). The number of patients demonstrating suppression on the 4-prism dioptre base-out test and the Worth 4 Dot test did not differ significantly. However, a larger proportion of patients exhibited suppression on the Bagolini test in the 0 < MRD1 ≤ 1 group than in the 1 < MRD1 ≤ 2 group (*p* = 0.02) (Table 2).

**Table 2:** Comparison of characteristics according to margin reflex distance 1.

	0 < MRD1 ≤ 1 (n = 19)	1 < MRD1 ≤ 2 (n = 26)	*p*-value
Magnitude of exodeviation (PD, mean ± SD)			
At distance	19.5 ± 6.3	20.9 ± 8.4	0.45
At near	22.1 ± 6.1	22.9 ± 7.8	0.48
Office based control score (mean ± SD)			
At distance	3.0 ± 1.65	2.6 ± 1.23	0.12
At near	1.9 ± 0.75	1.7 ± 0.69	0.23
Stereopsis (log arcsec, mean ± SD)			
Titmus test	2.36 ± 0.74	2.12 ± 0.70	0.22
Randot test	2.15 ± 0.37	1.87 ± 0.33	0.18
Distance stereo test	2.91 ± 0.43	2.79 ± 0.42	0.07
Presence of suppression, n (%)			
4PD base out test	13 (68.4)	7 (26.9)	0.11
Bagolini test	7 (36.8)	1 (3.8)	0.02
Worth 4 dot test	9 (47.4)	5 (19.2)	0.22

*MRD1* margin reflex distance 1, *PD* prism dioptres, *SD* standard deviation.

## Discussion

In this retrospective study, we compared the binocular function between the IXT-ptosis group and the IXT-only group. The mean distance stereoacuity, which was measured using the Distance Randot test, was significantly worse in the IXT-ptosis group. The number of patients exhibiting suppression on the Bagolini test was larger in the IXT-ptosis group than in the IXT-only group. The office-based control scores at distance and near were slightly worse in the IXT-ptosis group.

Congenital ptosis represents an eyelid malposition that can result in cosmetic, functional and psychosocial problems in children. It is commonly accompanied by significant ocular abnormalities or consequences^10^. It has been reported that the prevalence of amblyopia, strabismus, and refractive errors in patients with congenital ptosis is much higher than that in the general population^4–6,10,11^. Amblyopia has been reported to be more common in patients with congenital ptosis due to astigmatism or other refractive errors and/or obstruction of the visual axis^11^. Refractive errors such as astigmatism can cause image blur reducing stereoacuity. Regardless of myopia or hypermetropia, image blurring is proportional to the dioptric power of uncorrected astigmatism. Hence, stereoacuity worsens as the dioptric power of astigmatism increases^12^. In the current study, although patients in the IXT-ptosis group had slightly greater astigmatism, the difference between the two groups was not statistically significant (*p* = 0.07). Subjects in this study were corrected for “refractive error, if any” during stereoacuity and suppression tests. Thus, the effect of astigmatism on stereopsis could be ruled out.

Many studies have shown that patients with IXT have different degrees of damage to their binocular function^16^. Generally, near stereoacuity is normal in patients with IXT, although distance stereopsis can often be worse than in normal controls^14^. Other studies have suggested that some patients with IXT have a decline in near stereopsis^15^. Decreased distance stereoacuity likely represents poor control of the deviation and is often considered to be an indication for surgical correction. The near stereoacuity of the patients included in this study was not affected (mean ± SD = 2.13 ± 0.53 in the IXT-ptosis group; 1.93 ± 0.45 in the IXT-only group), and distance stereoacuity was significantly impaired when ptosis coexisted with IXT. Distance stereopsis can be more affected in patients with IXT ptosis because patients tend to look downward when they fixate at near. Furthermore, the office-based control scores at distance tended to be slightly worse in the IXT-ptosis group than in the IXT-only group, although this difference was not statistically significant.

Because the Bagolini test is performed without dissociation in a bright examination room and the Worth 4 Dot test has a greater degree of dissociation between the eyes than does the Bagolini test, the presence of suppression on the Bagolini test may indicate stronger suppression^16^. In this study, the number of patients showing suppression with Worth 4 Dot and 4-prism dioptre base-out tests did not differ significantly. However a larger proportion of IXT-ptosis patients had suppression on the Bagolini test than did the IXT-only group. One can infer that patients with deeper suppression are more likely to be in the IXT-ptosis group than in the IXT-only group.

Measuring the midpupil to upper lid distance (MRD_1_) is the best way to identify ptosis, even though the diagnosis is based on the patient’s clinical evaluation. It has been shown that when upper eyelidptosis assessment is conducted in a standardized manner, the interobserver and intraobserver variability are minimal and clinically acceptable^17^. None of the patients in the IXT-ptosis group had an MRD1 of less than zero because this study excluded patients with amblyopia. Compared with the 1 < MRD1 ≤ 2 group, patients in the 0 < MRD1 ≤ 1 group had worse distance stereopsis and a higher prevalence of suppression on the Bagolini test, indicating that stereopsis was more affected by severe ptosis.

Pressure from the ptotic eyelid on the corneal surface may cause astigmatism requiring close observation and optical correction. Surgical correction of ptosis is warranted if the astigmatism is significantly high. A chin-up position to be able to see beneath the droopy eyelids can cause further neck problems. Frontalis muscle contraction to further elevate the upper eyelid is a very common compensatory mechanism. This is also an indication for surgical correction^18^. Ptosis surgery can enable light to enter the eye and modify the corneal curvature, restoring corneal symmetry and reducing astigmatism; thereby changing the patient's refractive status^19^.

The severity of exotropia is determined by the magnitude of exodeviation, the ability to control the deviation and stereoacuity, which is a measure of the quality of binocular single vision^16^. Although there is no clearly established indication for surgical treatment in IXT, a worsening of the magnitude of exodeviation, motor control, or stereoacuity are some of the indications^20^. The results of this study reveal that binocular function is affected to a greater extent among IXT-ptosis individuals than among IXT-only patients. Whether this improves with ptosis correction needs further investigation. If the improvement of binocular function in IXT with ptosis patients after ptosis surgery is confirmed by additional study, early ptosis surgery should be considered in patients with IXT and ptosis even for mild ptosis.

This study has some limitations. This is a retrospective review of medical records, which could inherently limit the quality of data. The sample size was relatively small. This study does not disclose whether binocular function would improve after ptosis surgery. An adaptation issue may be caused by testing with a trial frame. This study excluded amblyopia and bilateral ptosis, and the external validity of the current study is limited to some extent. The strength of this study is the quantitative evaluation of binocular function in IXT patients with congenital ptosis compared to IXT-only patients.

In conclusion, the IXT-ptosis group had worse distance stereoacuity than did the IXT-only group, and a larger proportion of patients in the former group had suppression on the Bagolini test. Therefore, patients with IXT accompanied by ptosis had worse binocular function than did IXT-only patients. This should be assessed carefully and considered during treatment.

## Methods

### Study design and subjects

The medical records of 45 patients aged 4 to < 10 years who had been diagnosed with IXT accompanied by unilateral congenital ptosis (IXT-ptosis group) were reviewed retrospectively. The control group included 58 age-matched patients without congenital ptosis who were diagnosed with IXT (IXT only group). The study protocol adhered to the Declaration of Helsinki tenets and was approved by the Institutional Review Board of Yangsan Pusan National University Hospital (IRB number: 05-2020-267). Since this was a retrospective study, the need for informed consent of subjects was waived by the IRB.

Consecutive children aged 4 to < 10 years (at the first visit) with previously untreated IXT (other than refractive correction) and unilateral congenital ptosis who met the following criteria were eligible for this study: 1) intermittent exotropia at distance and either IXT or exophoria at near, 2) exodeviation with a magnitude of deviation measuring 15 prism dioptres or greater at distance or at near, based on measurements by the prism and alternate cover test (PACT). Congenital ptosis was defined as an upper eyelid with a margin reflex distance 1 (MRD1) measuring ≤ 2 mm. The exclusion criteria were as follows: 1) interocular difference in best-corrected visual acuity (BCVA) of ≥ 2 lines, 2) BCVA < 16/20 on either eye, or 3) presence of any ophthalmological abnormalities including optic nerve disease, or any other history of neurologic disease leading to a visual field defect.

### Clinical evaluations

Records of the patients were reviewed if they had undergone assessments of BCVA, stereoacuity, office-based control score, ocular alignment, suppression test, MRD1 and cycloplegic refraction at the first visit. Charts of all IXT cases with congenital ptosis during the time interval at one hospital were reviewed. Cases with missing information were excluded.

The BCVA was measured monocularly and followed the age-based testing protocol using Dr. Jin’s chart for children < 5 years of age and the ETDRS chart for older children. Dr. Jin’s chart consists of picture optotypes (car, airplane, duck, fish and butterfly) with a logarithmic progression of acuity levels, and it was designed to provide reliable visual acuity testing for children aged 4 to 7 years in South Korea^21^. The MRD1 is a measurement in millimetres from the light reflex on the patient’s cornea to the level of the centre of the upper-eyelid margin with the patient gazing monocularly in the primary position^22^. Patients with unilateral congenital ptosis (MRD1 ≤ 2) were included. MRD1 was measured when patients looked at the target monocularly because MRD1 can be affected by eyeball deviation.

Distance stereoacuity was assessed using the Distance Randot test at 3 m (m) and near stereoacuity was assessed using the Titmus stereoacuity test (circles, animals and fly) and the Randot Preschool Stereotest at 40 cm (cm). The eyelid was lifted up during stereoacuity testing when needed. Both Randot stereotests used in this study were random dot stereograms. The Titmus test had a local/contour stereo target^16^.

Office-based control scores were measured at a distance (6 m) and near (1/3 m) according to the classification of Kushner. Scores ranged from 1 (excellent, phoria) to 4 (poor, constant exotropia):^23^ 1-not tropic, recovers within seconds after cover; 2-briefly tropic only after cover, recovers in less than 5 s; 3- occasional spontaneous tropic, recovers with a blink; and 4-tropic frequently, constant exodeviation through a blink. In addition, ocular alignment was assessed using the cover/uncover test and the PACT at distance (4 m) and near (33 cm). The coexisting vertical deviation was defined as a constant deviation in all directions with a primary gaze angle of deviation of 4 prism dioptres or greater^16^.

The presence or absence of suppression was evaluated with the 4-prism dioptre base-out test, the Worth 4 Dot test and the Bagolini test. The 4-prism dioptre base-out test and the Bagolini test were assessed at 3 m. The Worth 4 Dot test was performed at 3 m and 33 cm^16^. During the assessment of stereoacuity, control score, the PACT and suppression, patients were corrected for BCVA.

### Outcome measures

The primary outcome measures were comparison of stereopsis and office-based control scores between the IXT-ptosis and IXT-only groups. The secondary outcome measures were comparison of stereopsis and office-based control scores between the patients in the 0 < MRD1 ≤ 1 and 1 < MRD1 ≤ 2 groups.

### Statistical analysis

Descriptive statistics for all patients were calculated for age, sex, BCVA, refractive errors, magnitude of deviation, stereoacuity, presence of suppression and office-based control scores. Pearson’s chi-squared test was used for categorical variables and the independent samples Student t-test was employed to compare continuous numerical variables. Before performing the t test, the normality of the data was assessed. All *p values* provided were obtained using a two-tailed test. SPSS for Windows 23.0 (SPSS Inc, Chicago, IL, USA) was used for all statistical analyses. Stereoacuity was converted into a log scale for analysis. For the purpose of statistical analysis, no measurable stereopsis was coded as 3.2 log seconds of arc^24^. Statistical significance was defined as a* p*-value smaller than 0.05.

## Data Availability

The datasets generated during or analysed during the current study are available from the corresponding author on reasonable request.

## References

[1] Rah SH, Jun HS, Kim SH (1997). An epidemiologic survery of strabismus among school-children in Korea. J Korean Ophthalmol Soc..

[2] McKean-Cowdin, R. et al. Multi-Ethnic Pediatric Eye Disease Study Group. Prevalence of amblyopia or strabismus in asian and non-Hispanic white preschool children: multi-ethnic pediatric eye disease study. *Ophthalmol.* **120**:2117–24 (2013). 10.1016/j.ophtha.2013.03.001 PMC4848013 23697956

[3] Srinagesh V, Simon JW, Meyer DR, Zobal-Ratner J (2011). The association of refractive error, strabismus, and amblyopia with childhood ptosis. J AAPOS..

[4] Anderson RL, Baumgartner SA (1980). Strabismus in ptosis. Arch Ophthalmol..

[5] Dray JP, Leibovitch I (2002). Congenital ptosis and amblyopia: A retrospective study of 130 cases. J Pediatr Ophthalmol Strabismus..

[6] Parsa CF, Robert MP (2013). Thromboembolism and congenital malformations: from Duane syndrome to thalidomide embryopathy. JAMA Ophthalmol..

[7] Govindan M, Mohney BG, Diehl NN, Burke JP (2005). Incidence and types of childhood exotropia: a population-based study. Ophthalmol..

[8] Han KE, Baek SH, Kim SH, Lim KH (2018). Prevalence and risk factors of strabismus in children and adolescents in South Korea: Korea National Health and Nutrition Examination Survey, 2008–2011. PLoS ONE.

[9] Lee DS, Kim SJ, Yu YS (2014). The relationship between preoperative and postoperative near stereoacuities and surgical outcomes in intermittent exotropia. Br J Ophthalmol..

[10] Nemet AY, Segal O, Mimouni M, Vinker S (2014). Associated morbidity of pediatric ptosis-a large, community based case–control study. Graefe’s Arch Clin Exp Ophthalmol..

[11] Wang Y, Xu Y, Liu X, Lou L, Ye J (2018). Amblyopia, strabismus and refractive errors in congenital ptosis: A systematic review and meta-analysis. Sci Rep..

[12] Kulkarni, V., Puthran, N. & Gagal, B. Correlation between stereoacuity and experimentally induced graded monocular and binocular astigmatism. *J Clin Diagn Res.* **10**:NC14–NC17 (2016). 10.7860/JCDR/2016/17341.7873 PMC4948432 27437256

[13] Kang, K.T. & Lee, S.Y. Relationship between control grade, stereoacuity and surgical success in basic intermittent exotropia. *Korean J Ophthalmol.* **29**:173–6 (2015). 10.3341/kjo.2015.29.3.173 PMC4446557 26028945

[14] Stathacopoulos RA (1993). Distance stereoacuity: assessing control in intermittent exotropia. Ophthalmol..

[15] Hatt, S.R. & Gnanaraj, L. Interventions for intermittent exotropia. *Cochrane Database Syst Rev.* **5**:CD003737 (2013). 10.1002/14651858.CD003737.pub2 16856017

[16] von Noorden GK : von Noorden’s BinocularVision and Ocular Motility. 5th ed, St. Louis, The CV mosby Co, 1996, pp.8–40, 182, 208, 209, 216, 218, 255, 265, 299, 301.

[17] Boboridis K, Assi A, Indar A, Bunce C, Tyers AG (2001). Repeatability and reproducibility of upper eyelid measurements. Br J Ophthalmol..

[18] Marenco M (2017). Clinical presentation and management of congenital ptosis. Clin Ophthalmol..

[19] Savino G (2016). Corneal topographic changes after eyelid ptosis surgery. Cornea.

[20] Lavrich JB (2015). Intermittent exotropia: Continued controversies and current management. Curr Opin Ophthalmol..

[21] Jin YH (1997). A new LogMAR vision chart: Jin’s Vision Chart. J Korean Ophthalmol Soc..

[22] Nerad, J.A. Evaluation and treatment of the patient with ptosis. In : Nerad JA, editor. Oculoplastic surgery: The requisites in ophthalmology. St. Louis: Mosby; 2001. p. 157–92.

[23] Kushner, B.J. Strabismus: Practical pearls you won't find in textbooks. Switzerland, Springer, 2017, chapter 6 exotropia, pp 73–96.

[24] Fawcett SL, Birch EE (2000). Interobserver test-retest reliability of the randot preschool stereoacuity test. J AAPOS..

